# Estimation of Incubation Period of Mpox during 2022 Outbreak in Pereira, Colombia

**DOI:** 10.3201/eid3001.221663

**Published:** 2024-01

**Authors:** Jorge M. Estrada Alvarez, Maryluz Hincapié Acuña, Hernán F. García Arias, Franklyn E. Prieto Alvarado, Juan J. Ospina Ramírez

**Affiliations:** Instituto Nacional de Salud, Bogotá, Colombia (J.M. Estrada Alvarez);; Salud Comfamiliar–Caja de Compensación Familiar de Risaralda, Pereira, Colombia (J.M. Estrada Alvarez);; Corporación Universitaria Minuto de Dios, Pereira (M. Hincapie Acuña);; SISTEMIC Research Group, Universidad de Antioquia, Medellin, Colombia (H.F. Garcia Arias);; Secretaria de Salud Pública y Seguridad Social, Pereira (J.J. Ospina Ramírez);; Instituto Nacional de Salud, Bogotá (F.E. Prieto Alvarado)

**Keywords:** mpox, monkeypox virus, infectious disease incubation period, person-to-person transmission, men who have sex with men, MSM, viruses, disease outbreaks, Colombia

## Abstract

We estimated the incubation period for mpox during an outbreak in Pereira, Colombia, using data from 11 confirmed cases. Mean incubation period was 7.1 (95% CI 4.9–9.9) days, consistent with previous outbreaks. Accurately estimating the incubation period provides insights into transmission dynamics, informing public health interventions and surveillance strategies.

Mpox, a zoonotic disease endemic to central and western Africa, is caused by monkeypox virus and transmitted primarily through person-to-person contact, secretions from skin and respiratory lesions, and fomites, such as bedding and shared utensils (*1*). Mpox manifests in a wide spectrum of signs and symptoms, including fever, headache, muscle pain, fatigue, lymphadenopathy, and a characteristic rash progressing from macules to papules, vesicles, pustules, and eventually crusts (*2*).

Since May 2022, mpox outbreaks have been reported in 109 countries, notably affecting men who have sex with men (MSM) (*3*). The atypical transmission pattern of mpox has prompted investigation into potential sexually associated routes and led to hypotheses about disease dynamics (*4*,*5*). Previous evidence suggests changes from classic clinical manifestations, which included milder symptoms and genital lesions, and alterations in transmission dynamics, particularly during the incubation period (*6*–*8*). 

Accurately estimating the mpox incubation period is crucial for implementing effective public health measures, such as quarantine and isolation during outbreaks. We attempted to estimate the distribution of the incubation period using data from the first confirmed cases among MSM identified through routine surveillance. This study was approved by the institutional committee of the Health Department of Pereira (Colombia) in accordance with Resolution 8430 of 1993. 

## The Study

We conducted a descriptive cross-sectional epidemiologic study using data extracted from field epidemiologic investigation of 11 reverse transcription PCR–confirmed cases of mpox in Pereira, Colombia, from the national reference laboratory of the National Institute of Health of Colombia. Case investigations were performed by a coinvestigator with training and experience in conducting epidemiologic interviews. Investigators gathered information from patients on travel history within the 21 days before symptom onset and any potential exposure events from a list of events developed from scientific publications on the 2022 mpox outbreak (*4*,*6*). Events included having multiple sexual encounters or new sexual partners, attending LGBTQ+ venues such as saunas and venues for sexual encounters involving chemsex (sexual activity while under the influence of drugs), or social gatherings involving intimate contact, including sexual activity. 

We used 2 approaches to collect data on date of exposure. If during the initial interview a case-patient reported an exact date potentially related to exposure to mpox infection, we recorded that as probable date of exposure. If the case-patient identified multiple potential exposure times, we recorded the longest period of time during which visits to places or situations for potential monkeypox virus transmission. That second approach generated censored interval data in which occurrence of the transmission event was known, but not the exact timing. 

For the date of onset, we used the day in the prodromal period on which symptoms were first reported, including nonspecific symptoms such as fever, fatigue, headache, lymphadenopathy, muscle pain, sore throat, or rash. We resolved inconsistencies in dates and missing data through follow-up telephone interviews. We conducted an event-time analysis to estimate the distribution that would best fit the incubation periods of the cases. Because measurements corresponded to both interval-censored and left-censored data, as explained elsewhere (*9*), we constructed a dataset with these characteristics. We used 3 parametric distributions—gamma, Weibull, and log-normal—to determine the best fit for the analytic model. We evaluated the fitted models using the corrected Akaike information criterion (AICc) to obtain the lowest value, which indicates a better model fit. We performed all analyses in R software icenReg package version 2015 (https://cran.r-project.org/web/packages/icenReg/index.html) using the maximum-likelihood method (*10*).

All 11 mpox case-patients included in our analysis were men who reported having sexual contact with other men. Median age was 34 (range 22–53, interquartile range 27–41) years. Symptoms commonly reported were myalgia, headache, fever, and lymphadenopathy. Only 1 case-patient did not manifest genital lesions, and only 2 did not have a previous HIV diagnosis. Dates of probable exposure for the 11 patients were July 9–September 10, 2022; dates of first symptom onset were July 11–September 20, 2022. Exposure window was 1 day in 6/11 cases; maximum exposure window was 8 days (1 case) and minimum 1 day. 

Visual inspection of the parametric curves confirmed that all 3 fitted parametric models provided reasonable fits for distribution of the incubation period (Figure). Based on AICc values (Table), Weibull parametric distribution (AICc = 61.18) fit the data most closely, followed by gamma (AICc = 61.64) and log-normal (AICc = 62.79) distributions. For the best-fit Weibull distribution, median incubation period was 7.1 days (95% CI 4.9–9.9 days); 95th percentile incubation period was 15 days (95% CI 10.6–22.6 days). Results for gamma and log-normal distributions were similar (Figure). Adjusted mean incubation period using the Weibull parametric distribution from likely exposure to onset was 7.1 days (5th–95th percentiles 1.9–15.0 days). That estimated incubation period aligns with some previous findings after considering associated uncertainty and statistical methodologies used in those studies. One study reported a mean of 8.5 days (5th–95th percentiles 4.2–17.3 days) slightly longer than in our study (*11*); another (K. Charniga, unpub. data, https://doi.org/10.1101/2022.06.22.22276713) estimated a mean 7.6 days (95% credible interval 6.2–9.7) and the 95th percentile 17.7 days (95% CrI 12.4–28.1 days), more closely consistent with our estimation and supported by data from 3 additional studies (*4*,*5*,*12*). 

**Figure:**
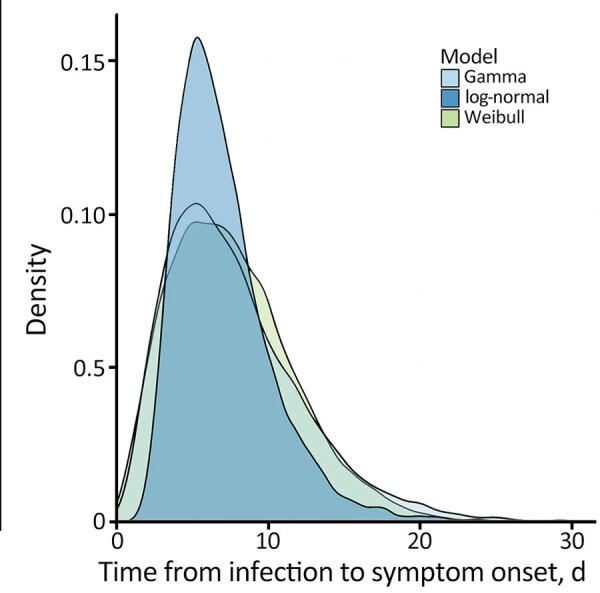
Comparison of parametric distributions from different models for the incubation period of mpox for patients in Pereira, Colombia.

**Table:** Estimated mean and percentile parametric distributions for incubation period in 11 confirmed cases of mpox, Pereira, Colombia*

Model	Mean incubation, d (95% CI)			AICc
	P_50_	P_5_	P_95_	
Gamma	6.7 (4.6–9.5)	2.0 (1.0–3.9)	15.9 (10.6–24.5)	61.64
log-normal	6.4 (4.3–9.2)	2.2 (1.0–3.9)	18.6 (10.7–37.6)	62.79
Weibull	7.1 (4.9–9.9)	1.9 (1.0–4.0)	15.0 (10.6–22.6)	61.18

*P_5_, P_50_, and P_95_ indicate the percentiles of the estimated distribution. AICc, corrected Akaike information criterion

Mpox might exhibit a shorter incubation period in invasive or complex exposures, in which the patient experienced contact through damaged skin or mucous membranes; typical incubation period is 9 days in those exposures (*4*). Mean mpox incubation period in MSM during this outbreak was 7.1 days, which aligns with reports in cases with similar conditions, falling within the typical values for complex or invasive exposure (*11*; K. Charniga, unpub. data). Of the 11 case-patients, 9 had a previous HIV diagnosis; research has indicated that HIV-infected patients may experience shorter incubation periods than persons not infected with HIV (*13*). That association might be related to immunosuppression and compromised immune response in persons with HIV potentially accelerating viral replication and clinical manifestations of mpox. 

## Conclusions

Establishing precise and accurate estimates of the temporal distribution of incubation periods for emerging infectious diseases is crucial for case definition and to inform public health policies. Robust inference methods that account for interval censoring in the estimations are recommended. Regarding potential transmission associated with sexual contact during the mpox outbreak (*6*), the complex and varied nature of those types of exposure should be carefully considered and incorporated into epidemiologic findings. We obtained the data on which this study was based from initial epidemiologic investigations of cases during a mpox outbreak in the city of Pereira, Colombia. The small sample size (11 case-patients) was a limitation of this study. 

Our study provides empirical evidence of the incubation period in the 2022 mpox outbreak, illustrated by the Weibull distribution graph, using data on exposure history and symptom onset in cases confirmed using PCR testing in Colombia and Latin America. The upper limit of the confidence interval for the estimated 95th percentile, 22.6 days, supports the recommendation of using a 21-day monitoring period for potential mpox cases involving close contact to limit further spread of the infection. Understanding the incubation period and its variability contributes to the development of targeted control strategies and enhances our knowledge of this emerging infectious disease. 
